# Membranoproliferative glomerulonephritis related to a streptococcal infection in a girl with IgA deficiency: a case report

**DOI:** 10.1186/s12882-020-01735-7

**Published:** 2020-02-27

**Authors:** Keisuke Sugimoto, Takuji Enya, Kohei Miyazaki, Tomoki Miyazawa, Tsukasa Takemura, Mitsuru Okada

**Affiliations:** 1 Department of Pediatrics, Kindai University Faculty of Medicine, 377-2 Ohno-higashi, Osaka-sayama, Osaka, 589-8511 Japan; 2 Department of Pediatrics, Kushimoto municipality Hospital, Wakayama, Japan

**Keywords:** IgA deficiency, Membranoproliferative glomerulonephritis, Streptococcus infection

## Abstract

**Background:**

IgA deficiency associated with glomerulonephritis is rare. In particular, there is no prior report regarding the association between IgA deficiency and membranoproliferative glomerulonephritis (MPGN) in children. Herein, we describe the case of a 5-year-old girl with selective IgA deficiency and MPGN.

**Case presentation:**

The patient presented with persisting urinary abnormality and hypocomplementemia following a group A treptococcal infection. Renal biopsy revealed the presence of diffuse mesangial hypercellularity, endocapillary proliferation, and focal thickening of the walls of the glomerular capillaries using light microscopy, with IgG and moderate C3 deposits observed using immunofluorescence. Electron microscopy images revealed nodular deposits in the subendothelial areas, with hump-shaped subepithelial deposits. The pathological diagnosis was confirmed as MPGN. Treatment using oral prednisolone (PSL), mizoribine (MZR), and angiotensin-converting enzyme inhibitors reduced the proteinuria. The PSL dose was gradually tapered, with the low dose of PSL and MZR continued for 4 years. Histological findings were improved on repeated renal biopsy, and PSL and MZR administration was discontinued.

**Conclusions:**

We report a rare case of MPGN related to a streptococcal infection in a child. The clinical presentation included selective IgAD, with several pathological findings and a clinical course typical of glomerulopathy. The patient was successfully treated using multidrug therapy.

## Background

Selective IgA deficiency (IgAD) is the most common primary immunoglobulin deficiency, identified in up to 1 per 600 individuals. The incidence of IgAD is lower in the Asian population, with an incidence rate of 1 in 14,840 to 1 in 18,500 in Japan [1]. Symptomatic patients with IgAD comprise approximately 10–15% of all patients with primary immunodeficiency [2], with most patients with IgAD being asymptomatic [3]. Those patients who are symptomatic, present with recurrent respiratory and gastrointestinal tract infections. There is also a high incidence of allergies, celiac disease, and autoimmune diseases, such as rheumatoid disease, systemic erythematosus, and thyroiditis, among patients with IgAD [4–6], with autoimmune diseases being more prevalent among adults (median age 29 years) and females with IgAD [7]. IgAD-associated glomerulopathies have also been reported, but are generally rare occurrences, with 8 cases of glomerulonephritis, including one case of membranoproliferative glomerulonephritis (MPGN), having previously been reported [8–13]. Uncommon pathological findings have been reported in patients with IgAD, with abundant deposits, on immunofluorescence (IF), of mesangial IgM (reported in 5 cases), and C3 (reported in 4 cases), except in the patient with MPGN.

## Case presentation

A 5-year-old Japanese girl was admitted to the hospital with a diagnosis of group A streptococcal infection, treated using antibiotics. Proteinuria and hematuria developed approximately 3 weeks after admission. She was transferred and admitted to our hospital for possible acute post-streptococcal glomerulonephritis, based on persisting urinary abnormality and hypocomplementemia. There was no family history of immunodeficiency and autoimmune renal disease.

On admission, there was an absence of oliguria, hypertension, and edema. Physical findings were as follows: height, 114 cm; weight, 20 kg; blood pressure, 98/50 mmHg; pulse, 88/min; and temperature, 37 °C. Urinalysis was significant, with a protein level of 100 mg/dL, moderate hematuria (50–99 red blood cells/high-power field) and mild proteinuria (0.3 g/day). Laboratory studies revealed the following: leukocyte count of 8600 mm^3^; red blood cell count of 399 × 10^4^ mm^3^; hematocrit of 32.2%; hemoglobin of 11.0 g/dL; platelet count of 35.7 × 10^4^ mm^3^; erythrocyte sedimentation rate (ESR) of 31 mm/h; and C-reactive protein level of 0.714 mg/dL. Electrolytes, serum creatinine, and blood urea nitrogen levels were normal. The creatinine estimated glomerular filtration rate (GFR) was normal (147 mL/min/1.73 m^2^). Aspartate aminotransferase (23 IU/L; normal range, 14–20) and alanine aminotransferase (13 IU/L; normal range, 10–40) levels also were within normal range, as were levels of lactate dehydrogenase activity and creatinine kinase activity. Serum immunoglobulin levels were as follows: IgG, 2300 mg/dL; IgM, 126 mg/dL; IgD, 15.6 (reference range, < 13 mg/dL); and IgE, 133 mg/dL. Of note, the serum IgA level was very low (< 25 mg/dL). Serologic analyses for antinuclear antibodies, SS-A, SS-B, anti-DNA, RNP, rheumatoid factor, and MPO-ANCA were negative. The serum level of complement component C3 was 73 mg/dL (normal range, 82–145 mg/dL) and 4 mg/dL (normal range, 12–33 mg/dL) for C4, with a total complement activity (CH50) of 10.8 U/mL (normal range, 24.2–52.8). The level of circulating immune complexes (CIC) was 5.5 g/mL (normal range, < 3.0 g/mL). The antistreptolysin O (ASO) level was 1362 IU/mL (reference range, < 239 IU/mL), with an antistreptokinase level of 2560 IU/mL (reference range, < 1280 IU/mL). Laboratory work-up for infection was negative for hepatitis B and C.

**Fig. 1:**
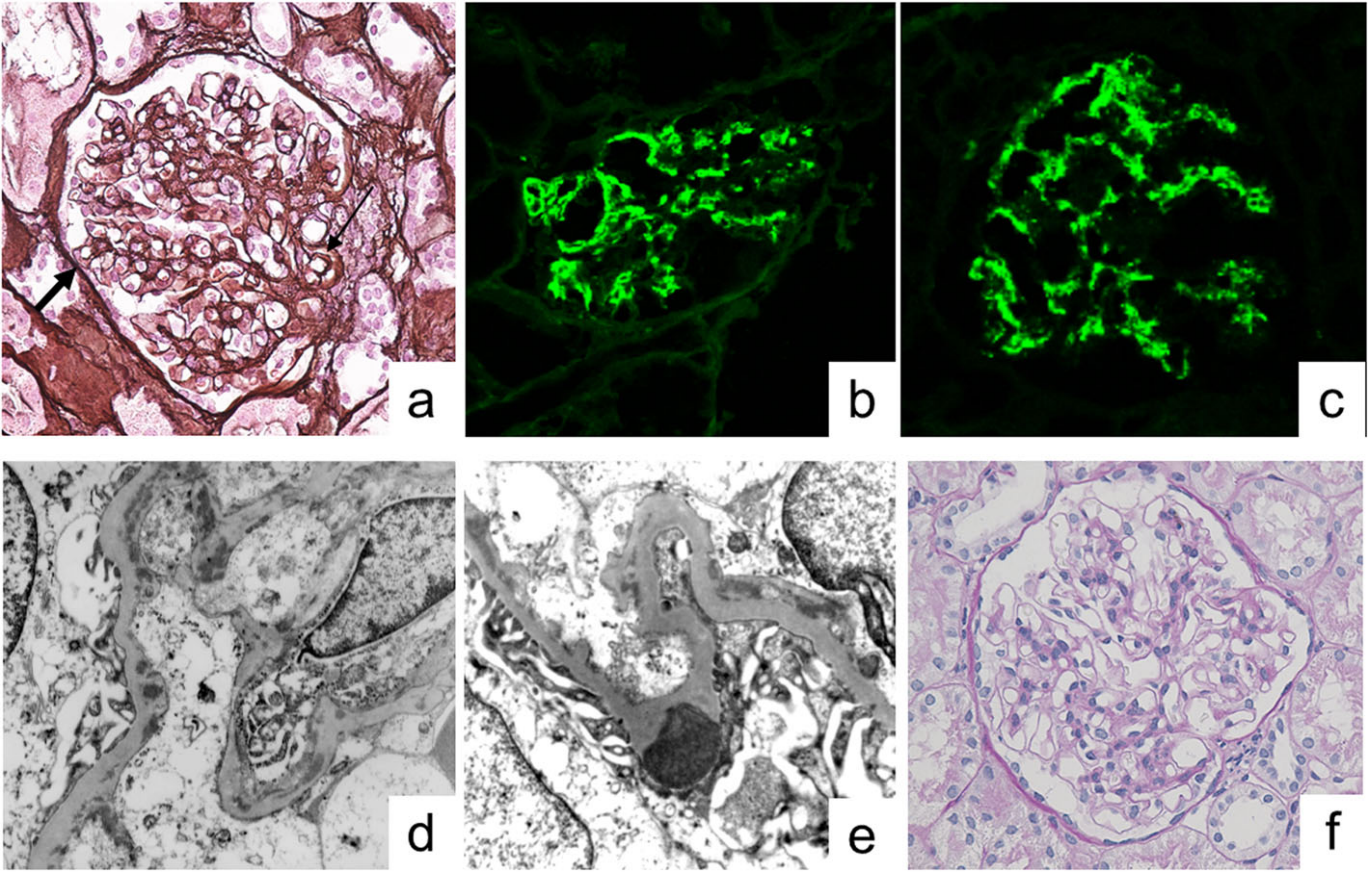
Kidney biopsy findings. **a** Light microscopy findings in a paraffin-embedded section. Diffuse mesangial proliferation, thick glomerular basement membranes (GBMs) and double contours of the GBMs (thin arrow) with mesangial interposition (thick arrow) are observed (periodic acid-silver methenamine staining, magnification 400×). **b-c** Immunofluorescence images, showing granular staining for IgG and C3 in the mesangial area and capillary walls. **d**-**e** Electron micrography images, showing marked subendothelial electron-dense deposits, including hump-shaped subepithelial deposits (magnification 6000×). **f** Absence of mesangial proliferation and GBM thickening (periodic acid-Schiff staining, magnification 400×)

On ultrasound examination, renal size and shape were normal, with absence of any mass lesion. A percutaneous kidney biopsy was performed on post-admission day 26 to determine the cause of persistent abnormal urine findings and hypocomplementemia. On histological examination of the renal biopsy specimen, 15 glomeruli were observed, with no evidence of sclerosis or crescents, but with diffuse mesangial hypercellularity, focal thickening of the wall of glomerular capillaries, and mesangial interposition (Fig. 1a). There was no evidence of cellular infiltration in both glomerular and interstitial areas, with absence of interstitial fibrosis, atrophy, or other injury of tubular cells, and necrosis. IF showed intense IgG and C3 reactivity in portions of the mesangium and glomerular capillary walls (Fig. 1b and c), but no reactivity for IgA and IgM, or other complement components, such as C4 and C1q. Staining for nephritis-associated plasmin receptor (NAPlr) was negative in the glomeruli. Electron microscopy (EM) images disclosed nodular deposits in the subepithelial areas (Fig. 1d). Hump-shaped subepithelial deposits were also observed (Fig. 1e). Taken together, these findings confirmed a diagnosis of MPGN induced by streptococcal infection.

**Fig. 2:**
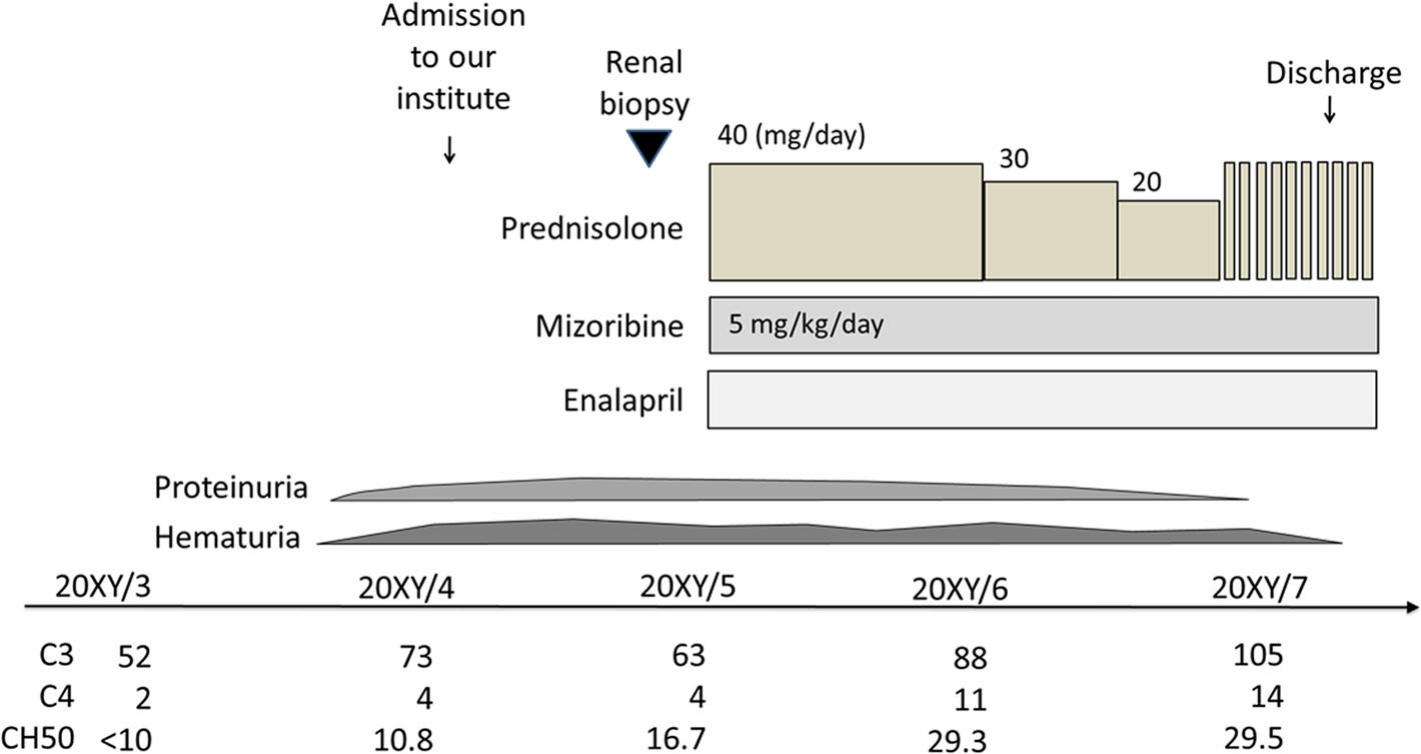
Clinical course. Multidrug therapy with oral prednisolone (40 mg/day), mizoribine (5 mg/kg/day), and enalapril were initiated. Consequently, urinary findings as well as serum concentration of complements improved. She was discharged after 2 months, and complete remission is maintained

Treatment was initiated using oral PSL (2 mg/kg/day), mizoribine (MZR; 5 mg/kg/day), angiotensin-converting enzyme (ACE) inhibitors (ACEIs (5 mg/day), and warfarin. Urinary findings gradually improved and complement levels normalized, with clinical remission achieved 4 months after treatment initiation. After tapering of PSL, low-dose PSL and other drugs were continued. A second renal biopsy was performed 4 years after the index admission to evaluate histological abnormality improvement. Histological examination revealed neither mesangial proliferation nor focal thickening of glomerular capillary walls. Mononuclear cell infiltration and fibrosis were also absent within the interstitium. PSL and MZR were discontinued, based on measured reduction in glomerular pressure, with only ACEI treatment continued (Fig. 2).

## Discussion and conclusions

In our case, the patient developed hematuria and proteinuria 3 weeks after the onset of a group A streptococcal infection. The renal biopsy revealed diffuse mesangial proliferation, with double contour of the glomerular basement membranes on light microscopy, and nodular deposits in subepithelial areas on EM, indicative of a MPGN lesion. Hump-shaped subepithelial deposits were also observed in our case, which provided additional evidence of streptococcal infection. Previous reports of IgAD with glomerulonephritis described several histological types, such as MPGN, focal glomerulonephritis, and glomerulonephritis. The following similar findings were identified using IF, namely IgM and C3 deposits within the mesangium and capillary walls, but without IgG [8]. A previous study indicated elevation of serum and secreted IgM levels in children with selective IgAD, but with normal levels of IgD [14]. These findings supported our hypothesis of a compensatory increase in IgM but not in IgD, resulting in the characteristic IF pattern observed. Interestingly, C3 and fibrinogen deposition, but not IgA, have been identified in skin biopsies of patients with Henoch-Schölein purpura associated with selective IgAD, despite the fact that pathological findings of Henoch-Schölein purpura are characterized by IgA deposition on the wall of the skin [12]. In our case, diffuse granular deposits of IgG and C3 in the area of the mesangium and glomerular capillary walls were present. Histological patterns of MPGN observed were consistent with previous reports of glomerulonephritis in IgAD. Although NAPlr was negative, elevation of the ASO titer, characteristic findings on IF staining, and hump formation on EM provided supportive evidence for a streptococcus infection-related nephritis (SIRN), accompanied by MPGN findings. CIC are detected in 50 to 60% of patients with selective IgAD and might be involved in the immunopathogenesis of vasculitis and glomerulonephritis [8, 15]. The presence of CIC for streptococcus antigen and antibody possibly induced MPGN in our patient.

A *good* prognosis of glomerulonephritis can generally be expected in most patients with IgAD without treatment, with no residual impairment in renal function [8]. Even in adults with IgAD-related mesangioproliferative glomerulonephritis, impairments in renal function are generally transient, recovering to normal levels with treatment using only angiotensin II receptor blockers [11]. We do note, however, one reported case of MPGN with IgAD in an adult, which rapidly progressed to end stage renal failure within 4 months, with the patient ultimately dying [13]. In contrast, a young adult man with SIRN (manifesting as MPGN), but not IgAD, presented with nephrotic syndrome that gradually improved, with complete remission achieved without treatment [16]. In our patient, complete remission was achieved at the early stage after treatment initiation, despite MPGN. There is a possibility of a SIRN diagnosis in our patient (which manifested as MPGN) and, thus, could have achieved remission without treatment. However, there is the possibility that a *good* therapeutic response and *good* prognosis are characteristic of MPGN with IgAD.

In conclusion, we report a rare case of MPGN associated with a streptococcal infection in a child. The findings from our case support that patients with selective IgAD can present with different pathological findings and clinical course of the associated glomerulopathy.

## Data Availability

Not applicable.

## Abbreviations

ACEI: Angiotensin-converting enzyme inhibitor
CH50: Total complement activity (CH50)
CIC: Circulating immune complexes
EM: Electron microscopy
ESR: Erythrocyte sedimentation rate
GFR: Glomerular filtration rate
IF: Immunofluorescence
IgAD: IgA deficiency
MPGN: Membranoproliferative glomerulonephritis
MZR: Mizoribine
PSL: Prednisolone
SIRN: Streptococcus infection-related nephritis
